# OVA: integrating molecular and physical phenotype data from multiple biomedical domain ontologies with variant filtering for enhanced variant prioritization

**DOI:** 10.1093/bioinformatics/btv473

**Published:** 2015-08-12

**Authors:** Agne Antanaviciute, Christopher M. Watson, Sally M. Harrison, Carolina Lascelles, Laura Crinnion, Alexander F. Markham, David T. Bonthron, Ian M. Carr

**Affiliations:** 1 ^1^Section of Genetics, Institute of Biomedical and Clinical Sciences, School of Medicine, University of Leeds and; 2 ^2^Yorkshire Regional Genetics Service, St James's University Hospital, Leeds, UK

## Abstract

**Motivation:** Exome sequencing has become a *de facto* standard method for Mendelian disease gene discovery in recent years, yet identifying disease-causing mutations among thousands of candidate variants remains a non-trivial task.

**Results:** Here we describe a new variant prioritization tool, OVA (ontology variant analysis), in which user-provided phenotypic information is exploited to infer deeper biological context. OVA combines a knowledge-based approach with a variant-filtering framework. It reduces the number of candidate variants by considering genotype and predicted effect on protein sequence, and scores the remainder on biological relevance to the query phenotype.

We take advantage of several ontologies in order to bridge knowledge across multiple biomedical domains and facilitate computational analysis of annotations pertaining to genes, diseases, phenotypes, tissues and pathways. In this way, OVA combines information regarding molecular and physical phenotypes and integrates both human and model organism data to effectively prioritize variants. By assessing performance on both known and novel disease mutations, we show that OVA performs biologically meaningful candidate variant prioritization and can be more accurate than another recently published candidate variant prioritization tool.

**Availability and implementation:** OVA is freely accessible at http://dna2.leeds.ac.uk:8080/OVA/index.jsp

**Supplementary information**: Supplementary data are available at *Bioinformatics* online.

**Contact:** umaan@leeds.ac.uk

## 1 Introduction

The application of next-generation sequencing for disease gene discovery or clinical diagnostics can generate large volumes of data, often resulting in identification of thousands of candidate disease genes or variants. A healthy individual genome can harbor more than a hundred genuine loss-of-function mutations (MacArthur *et al.*, 2012), making the identification of mutations responsible for a given phenotype a non-trivial task. As systematic experimental verification of each variant is infeasible, several computational prioritization methods have emerged in recent years that attempt to tackle this problem.

Candidate gene prioritization remains an active area of research despite a considerable number of algorithms and applications proposed in recent years (Adie *et al.*, 2006; Britto *et al.*, 2012; Chen *et al.*, 2009, 2011a,b). Thus far, while new methods and improvements continue to be introduced, there has been no universally applicable or precise approach. Classically, gene prioritization tools have been geared towards scrutinizing regional gene sets obtained from linkage studies. However, in recent years, next generation sequencing has become a *de facto* standard method for disease gene discovery in Mendelian diseases. Consequently, a few applications have recently emerged that expand and/or adapt currently used gene prioritization approaches to be more applicable for the evaluation of variants. For example, the Exomiser tool (Robinson *et al.*, 2014) supplements variant pathogenicity scoring with an algorithm for comparing human diseases with mouse phenotypes, while ExomeWalker (Smedley *et al.,* 2014) incorporates interactome data from STRING (Jensen et al., 2009). PriVar (Zhang *et al.*, 2013) combines variant pathogenicity scores from multiple sources together with pedigree information to rank variants.

Indeed, variant prioritization is not a novel concept—established algorithms like SIFT (Kumar *et al.*, 2009) and POLYPHEN (Adzhubei *et al.*, 2013) assess the likelihood of pathogenicity using information such as residue conservation status or the effects the change is likely to have on the protein. However, this approach is not without drawbacks—often variants predicted to be deleterious will produce no visible changes in phenotype due to redundancy in the genome.

Similarly, variant filtering approaches tailored to individual situations provide an alternative to pathogenicity scoring for reducing the size of candidate variant lists. Tools like AgileExomeFilter (Watson *et al.,* 2014) allow filtering of variants on a variety of criteria, such as inheritance mode, regions of autozygosity, sequencing quality or variant types thought to mostly be benign, for example synonymous substitutions or small in-frame insertions or deletions. However, as human exomes typically contain in excess of 30  000 variants, often neither approach proves adequate for pinpointing the correct mutation.

Here we investigate combining classic variant filtering techniques with a knowledge-based approach that utilizes data from multiple sources to prioritize variants. We take advantage of the success of the Open Biomedical Ontologies Foundry project (Smith *et al.*, 2007), which aims to standardize and create controlled vocabularies across multiple biological domains, and utilize data from multiple ontologies to facilitate computational analysis of natural language descriptions. Gene Ontology (Ashburner *et al.*, 2000) has received significantly more development effort than other OBO Foundry ontologies, and naturally a number of methods and applications that use GO annotations for candidate gene prioritization have been described in the literature (Chen *et al.*, 2011b; Eronen and Toivonen, 2012; Morrison *et al.*, 2005; Seelow *et al.*, 2008). However, so far little effort has been made to explore the potential of integrating of data from other ontologies for candidate gene prioritization. PhenoDigm (Smedley *et al.*, 2013), currently incorporated into Exomiser, utilizes The Human Phenotype Ontology (Köhler *et al.*, 2014) and Mammalian Phenotype Ontology (Smith and Eppig, 2009) to link human and model organism phenotypes. Similarly, the Phenomizer (Köhler *et al.*, 2009) tool takes advantage of The Human Phenotype Ontology to prioritize known disease genes in a clinical diagnostics setting.

Here, we integrate data from Gene Ontology(Ashburner *et al.*, 2000), The Human Phenotype Ontology(Köhler *et al.*, 2014), Uberon (Mungall *et al.*, 2012), UberPheno (Köhler *et al.*, 2013), Disease Ontology (Kibbe *et al.*, 2015) and The Pathway Ontology (Petri *et al.*, 2014) together with experimental interaction data from mentha (Calderone *et al.*, 2013) to develop a prioritization method that aims to increase the coverage, applicability and precision of currently available variant prioritization tools. The resulting application, ontology variant analysis (OVA), allows the user to control almost every aspect of the prioritization process, from custom phenotype selection and variant filtering to more in depth parameters, and is available at http://dna2.leeds.ac.uk:8080/OVA/index.jsp. We demonstrate the usefulness of our approach by testing OVA on multiple sets of candidate disease genes and exomes, encompassing known and novel disease genes. Finally, we show that our tool compares favorably to another recently published variant prioritization tool, ExomeWalker (Smedley *et al.*, 2014).

## 2 Methods

**Fig. 1.**
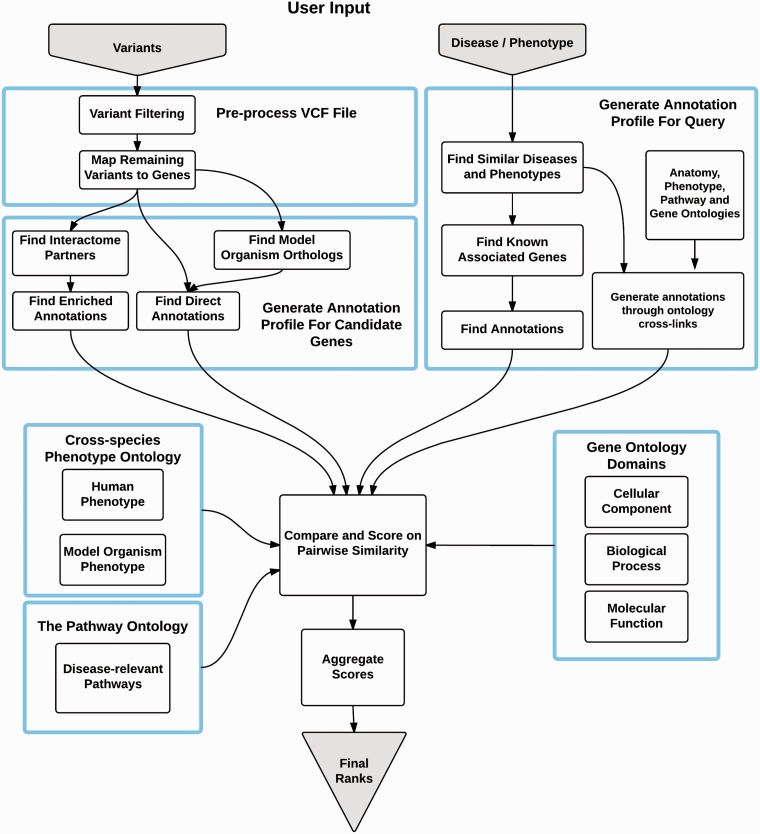
Overview of OVA workflow

OVA is aimed at prioritizing data from whole exome sequencing experiments and the workflow consists of three main steps, summarized in Figure 1. Uploaded VCF (variant call format) files are passed through custom user filters in order to substantially reduce candidate search space by removing likely benign variation. Each remaining variant is mapped to a gene for which an extensive multi-ontology annotation profile is derived from direct annotations and supplemented with inferred annotations from model organism data and data from the local interactome neighborhood.

For a given query phenotype or disease, a comparison annotation profile is computed from known phenotype-genotype associations, phenotype similarities and cross-links between multiple ontologies to infer new knowledge.

OVA compares each candidate to the phenotype/disease annotation profile and calculates a series of similarity metrics. These scores, together with a number of other related features, are used as an input to a model built using a supervised learning approach, which calculates the probability of each candidate gene harboring the disease-causing variant.

### 2.1 Variant filtering

OVA allows users to select variant filters which are relevant to their own data. These include variant classes which are often deemed benign variation, such as synonymous single base substitutions, small in-frame insertions or deletions or variation within intronic and/or untranslated regions. Additionally, a chromosomal region filter can be used where, e.g. autozygosity mapping data is available.

Genotype filtering integrates support for multi-sample VCF files. This can be used in a number of ways. For instance, in a case of an autosomal recessive phenotype, only variants which are homozygous or compound heterozygous in the affected but not the unaffected patient samples can be retained. Any genotype combination is permissible, allowing the user complete control of this step.

A number of additional filtering options, such as quality score and allele frequency in Born in Bradford project data, are also included. It is also worth noting that the variant filtering approach implemented in OVA uses transcript, rather than gene, sequences in order to take codon position plurality (Subramanian, 2015) into account.

### 2.2 Measuring semantic similarity between ontology terms

In recent years, ontologies have become a *de facto* standard for organizing knowledge in the biomedical domain in a structured manner. An ontology is a directed, acyclic graph in which vertices correspond to terms and edges represent the relationships between terms. Ontology terms are organized in a hierarchical manner, with broad terms nesting towards the root of the ontology, while more specific terms are further away from the root. Collectively, the terms represent a controlled vocabulary describing a particular domain, and this structure can facilitate computational analysis of entities and concepts annotated with the terms. Ontological annotations largely circumvent the problems that arise from the use of natural language descriptions, such as ambiguity and subjectivity, and have been invaluable in organizing data in large scale genome annotation projects (Legaz-García *et al.*, 2012).

The structure of an ontology and corresponding annotations allows the computation of semantic similarity between entities such as genes or diseases. Although gene similarity has been classically compared using sequence similarity (an evolutionary measure), the strength of semantic similarity is that the comparison is driven by the meaning of the descriptions pertaining to each gene. For example, simple lexical comparison of the words ‘*foal’* and ‘*horse’* would classify them as unrelated. Similarly, sequence-based comparisons will tell us nothing about the similarity of two genes which differ in sequence greatly, but perform key functions in the same biological process or pathway.

In order to quantify the semantic similarity between entities, one must first quantify the similarity of terms used to describe them. Semantic similarity between two terms, *a* and *b*, can be described as the amount of information shared by the two terms. Given a hierarchical ontology structure, this can be quantified thus:

(1) $$\text{Sim}\left(a\text{, }b\right)=\frac{{\text{IC}}_{\text{MICA(a, b)}}}{{\text{max\{IC}}_{\text{a }}{\text{, IC}}_{\text{b}}\text{\}}}$$

where IC_MICA(a,b)_ is the information content (IC) of the most informative common ancestor (MICA) of the terms a and b. In information theory, the IC of a term *t* can be given as:

(2) $${\text{IC}}_{\text{t}}=\;-\text{ln(}P_{\text{t}})$$

where *P*_t_ is the probability of observing the term in a gold standard corpus. UniprotKB (Magrane and Consortium, 2011) annotations is a frequently used corpus for estimating IC of Gene Ontology terms and has been shown to facilitate robust semantic similarity measurements (Pesquita *et al.*, 2009b). However, the annotation corpora that could be used to calculate the IC of terms within other biomedical ontologies are rarely complete or bias-free. Furthermore, there are a number of issues that can arise when estimating IC using a corpus, including bias towards the better characterized concepts, ‘orphan’ terms and the variability of the measure due to the evolution of the corpus (Lord *et al.*, 2003). Thus, in order to accurately quantify similarities between terms within multiple ontologies, a different approach is needed. Here, we modify a previously described topology-based measure (Mazandu and Mulder, 2012).

The probability of occurrence of a term can be estimated using the intrinsic ontology structure—terms further away from the root are expected to be more specific and thus occur less frequently. For example, The Human Phenotype Ontology term ‘*Abnormality of the eye’* (HP:0000478) is less informative than the term ‘*Glaucoma*’ (HP:0000501), its descendant.

The level of the term in the ontology graph does not always correlate with its specificity. However, a number of topological characteristics in the ontology graph can help correct where this is not the case. The number of direct descendants of each term can be interpreted thus: if a term has a large number of children, its children are more specific than those of a term that has fewer children, as it encompasses more branches in the sub-domain. Furthermore, parents and their positions within the ontology graph should be taken into account. A term that descends from highly specific parent terms can be reasoned to be more informative than a term descending from less specific parent terms. The original approach (Mazandu and Mulder, 2012) considers the specificity of all direct ancestors of a term, using a product formula to calculate probabilities of occurrence, which, in particular in ‘deeper’ ontologies, can result in the inflation of specificity of terms with more than one direct ancestor; and this propagates down the ontology tree. In our approach, we consider only the most informative direct ancestor of a term in order to model a lower rate of IC gain while traversing down the ontology tree.

An ontology is never cyclic—thus, while a term may have multiple parents, it is impossible for a term to have a parent that is also its descendant. However, multiple direct ancestors of a given term may have child-parent relationships of their own. This property of the ontology graph allows calculating the IC of each term recursively, starting from the root of the ontology:

(3) Pt= {1if rootPaCa ·0.2if  t   'is_a' aPaCa ·0.4if  t  'part_of' a

Where *P*_a_ is the probability of occurrence of a direct ancestor of term *t* and *C*_a_ represents the number of children direct parents of term t have. We only consider ‘is_a’ and ‘part_of’ relationship types in all ontologies, giving more weight to ‘is_a’ type edges. Thus, using this approach, the similarity of a term to itself is 1, as the MICA of a term and itself is itself. Similarly, because the IC of the root is −ln (1) = 0, any two terms for which the only common ancestor is root will have a similarity of 0. This definition defines a normalized range of semantic similarity for two terms.

The similarity measure described here was thus applied to the three domains of the Gene Ontology, The Human Phenotype Ontology, Uberpheno, Uberon and The Pathway Ontology, with IC of each term, and pairwise similarities between all terms within each ontology stored in an intermediate MySQL database.

Semantic similarity between two entities—genes or diseases—is calculated from pairwise ontology term similarities. An entity is rarely annotated with just a single term—thus, a measure to combine pairwise similarities into a single score is needed. Although three approaches are frequently used in the literature (Pesquita *et al.*, 2008)—the average, maximum or best match average of pairwise similarities- here we use the best match average approach, as this provides the highest score resolution (Pesquita *et al.*, 2009a).

Pairwise semantic similarity approaches for gene semantic similarity can suffer from a bias arising from ‘shallow’ annotations. Although a pair of terms deep within the ontology separated by the same distance will have higher semantic similarity than those closer to the root, the semantic similarity between a term and itself is always 1. Thus, two highly functionally divergent genes could contain a high-level annotation such as ‘protein binding’ and the resulting match would lead to an increase in the final pairwise score which may bias the results. We attempt to address this issue by taking into consideration the IC of a term where terms being compared are equal. We modify the frequently used best match average approach to adjust perfect matches to contribute less to the final score if they are not informative, and more if they are.

### 2.3 Scoring

Initially, we derive an annotation profile of functions, processes, cellular and anatomical components, pathways and model organism phenotypes that may be relevant to the query human disease. This is accomplished in two ways. First, using phenotype semantic similarity, we select all genes which have been previously linked to diseases presenting similar phenotypes to the query. We use direct gene annotations as part of the annotation profile. Second, given a query disease, we can derive annotations by reasoning across ontologies, starting from phenotype terms. For example, UberPheno phenotype term ‘*abnormal(ly) disrupted determination of left/right symmetry*’ (ZP:0000333) can be directly linked to GO Biological Process term ‘*determination of left/right symmetry’* (GO:0007368); similarly, Uberon anatomical ontology term ‘*heart*’ (UBERON:0000948) can be linked to multiple Gene Ontology terms such as ‘*heart morphogenesis*’ (GO:0009653), phenotype terms such as ‘*Cardiomegaly*’ (HP:0001640) and pathway ontology terms such as ‘*cardiovascular system disease pathway*’ (PW:0000020).

For each candidate gene, we compare direct ontology annotations to the disease annotation profile. Using the semantic similarity measure described above, similarity scores are computed in every domain for each candidate gene.

One of the major hurdles to overcome in a knowledge-based approach to candidate gene prioritization is the inconsistency of the level and quality of annotations across the genome. Although the better studied genes are more likely to have high quality annotations, less well characterized yet more relevant genes can be overlooked simply because information available about them is incomplete. We aim to address this issue in OVA using two different approaches. We use annotations from model organism (mouse and fish) orthologs to both support the human data and to compensate where data for human genes remains incomplete. Furthermore, for each candidate disease gene, we consider the neighborhood within the human interactome. Interacting groups of proteins are more likely to participate in the same or similar processes, and thus, if a protein lacking in quality functional annotations is known to interact with a group of proteins for which informative annotations are available, these can be extended to apply to the poorly annotated gene.

We define the interactome neighborhood as a set of genes sharing direct interactions with the gene in question. These are derived from mentha (Calderone *et al.*, 2013), a collection of curated physical protein-protein interactions from several primary databases. Here, we use a gene set enrichment approach to select only annotations which are over-represented in the interactome neighborhood in order to reduce noise and extract common functions. A Fisher’s exact test was used to test for term enrichment within the interactome neighborhood against whole interactome background. Bonferroni correction (Armstrong, 2014) was applied to account for multiple testing. Terms with corrected *P* < 0.01 in the interactome neighborhood are retained for comparison.

We consider and compare three different approaches to arrive at final aggregate ranks: the average of similarity scores across all domains; a weighted average; and a supervised learning approach.

The weighted average approach gives more weight to features which can be considered more informative—e.g. while information about cellular localization of a protein is important, a mouse model with a highly similar phenotype to query disease is a much stronger predictor of a good candidate disease gene. Additionally, this approach can dynamically adjust the similarity score weights based on information available about the candidate gene. A low score of a gene poorly annotated in a particular domain is not always comparable to a low score derived from multiple informative annotations.

In order to find an optimum scoring function for the multiple derived metrics, we consider a supervised learning approach. We use two-thirds of all OMIM diseases with at least one known causative disease gene to create a training dataset and use our scoring functions to assign a semantic similarity score for each category for each known disease gene and a matched randomly selected gene. We label each instance as either ‘disease gene’ or ‘non-disease gene’ and consider each score as a feature to be learned. Furthermore, we also consider a number of other features for each instance, such as the number and informativeness of annotations that support each score; proximity in the interactome to known disease genes; disease type.

We use an established data mining java package WEKA (Hall *et al.*, 2009) to train a Random Forest (RF) model—ensemble learning algorithm which constructs and combines information gained from multiple decision trees and is robust to overfitting.

Given a training data set consisting of *n* instances of genes, let *V*_i_ = {*f*_1_…*f_m_*} be a feature vector describing the *i*th gene and *L*_i_ its class label (‘disease gene’ or ‘non-disease gene’). Briefly, in a RF model, each decision tree is constructed using a bootstrapped sample of instances from the training data *n* and a randomly selected subset of features *f,* consisting of *x* < *m* features. In a decision tree, a set of rules describing *L* are learned from the training data by recursively splitting the feature space at each node until all leaf nodes contain instances from only one L class. Given an unknown instance, each tree in an RF model ‘votes’ for the likeliest class label, with the percentage of individual trees voting for a given class representing the posterior probability that the instance belongs to that particular class. For more details on RF models, see (Breiman, 2001).

We find that the optimum performance/accuracy trade off was reached with a model of 600 decision trees, each considering *x* = 6 random features. We pre-process the training dataset by removing misclassified instances using 10-fold cross-validation of the original model, as these likely represent outliers, and use it build the final model. We classify each candidate gene using our model and use classification prediction distribution to obtain the likelihood for the class labels being correct. We use this confidence value for ‘disease gene’ class to obtain final ranks for candidate genes.

### 2.4 Testing datasets

In order to assess the performance of OVA, we have used multiple datasets. Initially, all OMIM (Online Mendelian Inheritance in Man, http://www.omim.org/) and Orphanet disease entries with known molecular basis were selected, comprising 1340 disease/gene combinations for which two or more known causative genes have been attributed and 2964 disease/gene pairs with only one known causative gene. We use two thirds of these to train our model as described above, while the remaining disease/gene pairs form testing **Datasets 1** and **2**.

**Dataset 1** consists of all OMIM or Orphanet disease entries not used for training with at least two known causative genes attributed. This dataset comprises of 442 disease-gene sets and aims to simulate use cases where a novel disease gene causes a disease with a previously described genetic basis.

**Dataset 2** consists of all OMIM or Orphanet disease entries not used for training where only one known causative gene is known. This dataset comprised 978 disease-gene sets and aims to simulate use cases where a novel disease gene causes a disease with no previously known genetic basis.

All VCF files used for testing were generated by simulating the presence of a single known deleterious variant into one of our in-house exomes from healthy individuals.

**Dataset 3** consists of 150 VCF files, each containing a known deleterious variant from ClinVar (Landrum *et al.*, 2014) database. These were selected on the following criteria:

- Annotated as ‘Pathogenic’
- An insertion, deletion or single nucleotide substitution
- Annotated to an OMIM disease with at least one other known disease gene

**Dataset 4** consists of 20 VCF files, each containing a deleterious variant that has been published since the beginning of January 2015. These variants are summarized in Supplementary Data S1.

## 3 Results

We use **Dataset**s** 1** and **2** to assess OVA performance for simulated disease cases both with and without a previous known molecular basis. For each disease gene tested in **Datasets 1** and **2**, we remove any known disease or human phenotype associations in our database, in order to simulate cases of novel gene discovery. Human phenotype-related features that directly link the test gene to disease are also replaced with unknown values in the instance classification step. We rank each test gene with respect to disease together with 200 randomly selected genes from the pool of all human genes which have at least minimal Gene Ontology annotations in order to avoid any bias, as known disease genes are rarely entirely unannotated. We assess three methods for obtaining the final scores, as discussed in the Methods section—average, a weighted average approach and a machine learning approach.

**Fig. 2:**
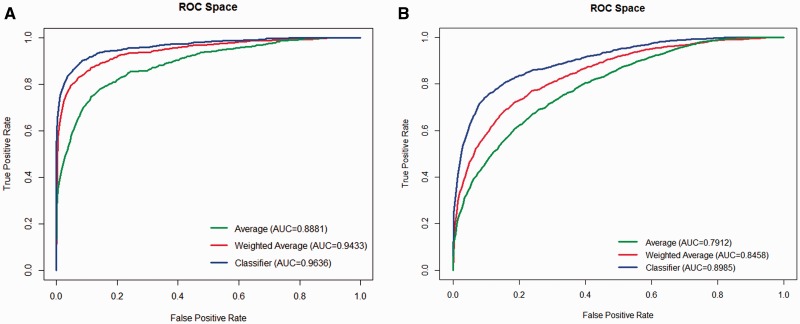
ROC curves showing the performance of OVA while using Dataset 1 **(A)** and Dataset 2 **(B).** Each dataset was prioritized using three methods—the average (green), the weighted average (red) and a classifier (blue) built using a supervised learning approach

Receiver operator characteristic (ROC) curves and corresponding area under the curve (AUC) values were calculated using R package ‘ROCR’ (Sing *et al.*, 2005). Figure 2A shows the ROC curves obtained using Dataset **1**, while Figure 2B shows the ROC curves obtained using Dataset **2**. There is a notable difference in performance between the three methods that is consistent across the datasets used. Our model, which was trained using 32 features, is able to prioritize candidate disease genes with greater accuracy than the average or a weighted average approaches using the same scores.

As expected, there is a notable difference in performance between **Dataset**s** 1** and **2**. Performance of OVA is greatly enhanced (AUC up to 0.9636) where knowledge about previously identified molecular causes of the disease is available. However, extending the search to diseases which cause similar phenotypes allows the prioritization of cases where little is known about the molecular causes of a disease that is still robust (AUC up to 0.8985).

The key parameter in OVA is the phenotype filter. In order to ascertain how sensitive our algorithm is to various amounts of input noise, we have used **Dataset 1** to simulate cases where the phenotype is inaccurately or inadequately described. Introducing additional irrelevant query phenotypes had less of an impact on the accuracy of the results than excluding relevant terms (Supplementary Fig. S1A and B); however, our method tolerates minor levels of noise. OVA incorporates an extensive variant filtering step available for VCF files, including support for multi-sample VCF files with multiple affected/unaffected patients that can be often available to researchers studying rare genetic diseases. As such, we compared variant prioritization capabilities of OVA with another recently reported variant prioritization tool, ExomeWalker (Smedley *et al.*, 2014) which uses a current state-of-the-art algorithm for network-based gene prioritization, coupled with a variant scoring approach. We use **Dataset 3** to see how these two tools compare. In order to simulate novel gene discovery, we supply all known genes for the disease (except the test gene) as seeds to ExomeWalker; for OVA, any associations between test gene and disease are removed as previously described. For both tools, we provide the inheritance mode of the disease for each test instance and use default parameters.

**Fig. 3.**
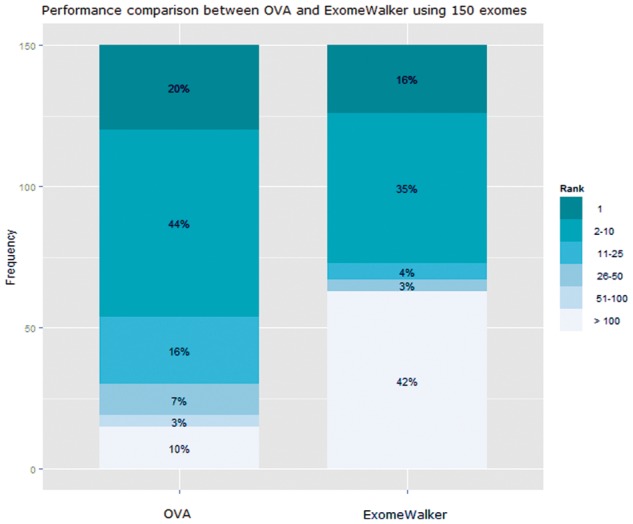
Performance comparison between OVA and ExomeWalker using 150 exomes

Figure 3 shows the disease gene rank distributions obtained by both tools. Out of 150 VCF files, in 20% of the cases OVA ranked the true disease gene first, with ExomeWalker performing similarly at 16%. A total of 64% of instances were ranked in the top 10 by OVA, compared with 51% by ExomeWalker. Although ExomeWalker scored 58% of all cases very accurately and the other 42% poorly, only 10% test cases were ranked outside the top 100 by OVA.

Additionally, as OVA can be used to prioritize genes as well as variants, we have opted to compare our approach with another gene prioritization tool, G2D (Perez-Iratxeta *et al.*, 2007). G2D can prioritize candidates using data from GO annotations, sequence similarity, MeSH terms and STRING protein-protein interactions. We have chosen this tool for comparison due to parallels in data sources used by OVA; use of ‘seed’ gene input which enables a cross-validation based testing approach that we applied to OVA and ExomeWalker; and finally, a straight forward web application that facilitates automation for large testing data sets. Using **Dataset 1**, we provided as input to G2D a 100 MB genomic region containing a test gene and other known genes associated with the disease as seeds, and similarly prioritized all genes within the same region with OVA. Although OVA significantly outperformed G2D (AUC 0.9593 versus 0.8524, Supplementary Fig. S2), some of the differences in accuracy could be accounted for by somewhat outdated data used by G2D (as of this writing, last updated in 2010).

Finally, in order to verify that our results are consistent with real cases of novel disease gene discovery, we use our model to prioritize 20 recently published novel disease gene mutations (**Dataset 4**). We use a version of our database frozen before publication dates to assess performance. Table 1 summarizes the results. The ranking is overall somewhat poorer than that observed using our other datasets, with 14 out of 20 genes ranked in the top 25.

**Table 1.** The prioritization results of Dataset 4, consisting of 20 novel disease gene VCF files (see Supplementary Data S1 for details)

Ranking	Count	Percentage (%)	Genes
**First**	3	15	CACNA1B, COQ4, WWOX
**Top 10**	8	40	CACNA1B, COQ4, WWOX, KCNA2,
			NALCN, SEMA3D, SLC9A1, USP8
**Top 25**	14	70	CACNA1B, COQ4, WWOX, KCNA2,
			NALCN, SEMA3D, SLC9A1, USP8,
			AFF4, DCDC2, DTNA, ETV6,KCNC1, PNKP
**Not in top 25**	6	30	CHCHD10, COL17A1, DDX58,
			PTRH2,SNRPB, CEP120

## 4 Discussion

The development of open biomedical ontologies has exploded in the last decade and alongside it the coverage and accuracy of annotations. Here we take advantage of this rich resource to bring together ontologies from across multiple domains to produce OVA, a knowledge-based gene and variant prioritization tool. OVA utilizes human and model organism phenotypes, functional annotations, curated pathways, cellular localizations and anatomical terms to find genes most relevant to a query phenotype.

OVA exploits The Human Phenotype Ontology and Uberpheno structure and annotations to facilitate comparisons between human diseases and animal models. Terms pertaining to model organism phenotypes [e.g. ‘*Abnormal snout morphology’* (MP:0000443)] are bridged to human phenotype terms [e.g. ‘*Abnormality of the nose*’ (HP:0000366)] by Uberpheno, allowing us to quantify similarities between them from the ontology graph. As numerous large scale phenotyping efforts are currently under way, such as those undertaken by The International Mouse Phenotype Consortium (Skarnes *et al.*, 2011), utilizing model organism phenotype data in a generalized gene prioritization approach is becoming more viable as coverage increases.

Gene ontology annotations have proved to be one of the most frequently utilized sources of gene functional knowledge in computational biology, with numerous applications taking advantage of this structured and highly curated resource. GO has also been heavily utilized as a data source for various candidate disease gene prioritization applications. However, while candidate prioritization methods using Gene ontology semantic similarity measures have generally been demonstrated to be effective, there are a number of drawbacks that this type of methodology suffers from that can detract from usability, accuracy and utility.

The majority of tools catalogued at Gene Prioritization Portal (Bornigen *et al.,* 2012) require the user to supply ‘seed’ genes—genes already known to be associated with the disease—and score candidates based on similarity to these. This is a major limitation of this approach, as in the case of rare or novel phenotypes, any prioritization based on similarity to known disease genes is impossible. Furthermore, the quality of available annotations of the supplied genes largely determines the success of this type of approach, while also allowing for little functional heterogeneity among disease genes.

Here, we supplement this approach by building links across multiple ontologies. This allows enhancing the functional profile against which all candidate genes are scored by reasoning directly from a disease phenotype, as well as known genes. Consequently, this approach eliminates the requirement for the user to supply known ‘seed’ genes and reduces the reliance on quality seed gene annotations.

The integration of data from across multiple ontologies can supplement knowledge where it may be incomplete or inadequate in a particular domain. Although most human genes now have GO annotations available, ‘shallow’ annotations—i.e. low IC terms—are still prevalent. Similarly, there are a number of terms that while they may not be considered uninformative, are not meaningful for candidate gene prioritization without further context. Two genes annotated with the term ‘*regulation of transcription, DNA-templated*’ (GO:0006355) would be considered highly functionally similar, and yet could participate in regulation of entirely different pathways. Accordingly, pathway ontology annotations can serve to fill in this knowledge gap, helping to decide whether a gene is truly relevant to the query phenotype and improving prioritization accuracy by removing noise.

Here, we simulate novel gene discovery in well and poorly characterized diseases and show that our method is capable of meaningful candidate gene prioritization even when direct functional knowledge about the disease is lacking (Fig. 2). By inferring new gene-disease associations through phenotype semantic similarity search and cross-ontology bridges, OVA attempts to deduce missing annotations, enabling gene prioritization for new and rare human diseases and supplementing the functional profile of better characterized phenotypes. We show that our model distinguishes relevant genes accurately and, coupled with our variant filtering approach, performs better than another recently published variant prioritization tool (Fig. 3).

Knowledge-based candidate gene/variant prioritization methods have been known to perform worse than reported when predicting novel disease genes (Bornigen *et al.*, 2012). However, large scale assessment using novel disease genes is not feasible. Cross-validation-based methods of individually removing direct disease-gene associations can serve to simulate novel gene discovery by ensuring that the test gene does not contribute to the query annotation profile. However, there is a degree of knowledge circularity in ontology annotations that is difficult to account for. By prioritizing VCF files containing 20 newly reported mutations in novel disease genes, we show that there is agreement between these results and those obtained from a larger, simulated dataset (Table 1 and Fig. 3), although the novel variant dataset prioritization was somewhat less accurate. Thus, we maintain that our results on previously described disease genes represent a reasonable approximation of the true accuracy of OVA.

Through an interactive and intuitive web interface, OVA allows the user to control many aspects of the prioritization process. OVA employs The Human Phenotype Ontology to facilitate detailed phenotypes queries in addition to previously described diseases, enabling prioritization for novel diseases. OVA can be accessed freely at http://dna2.leeds.ac.uk:8080/OVA/index.jsp through any modern browser with JavaScript support.

## Supplementary Material

Supplementary Data

## Notes

*Funding.* This work was supported by grants from Sir Jules Thorn Charitable Trust (Grant 09/JTA) and the Medical Research Council (MR/L01629X/1).
