# Supplementary material for: OVA: integrating molecular and physical phenotype data from multiple biomedical domain ontologies with variant filtering for enhanced variant prioritization
Source: Bioinformatics. 2015 Aug 12;31(23):3822–9. doi: 10.1093/bioinformatics/btv473 (PMC4653395; doi:10.1093/bioinformatics/btv473)
Supplement: Supplementary Data [file supp_31_23_3822__index.html]

OVA: integrating molecular and physical phenotype data from multiple biomedical domain ontologies with variant filtering for enhanced variant prioritization — OVA: integrating molecular and physical phenotype data from multiple biomedical domain ontologies with variant filtering for enhanced variant prioritization — Supplementary Data 

# OVA: integrating molecular and physical phenotype data from multiple biomedical domain ontologies with variant filtering for enhanced variant prioritization

## Supplementary Data

files

- Supplementary Data - xlsx file
- Supplementary Data - png file
- Supplementary Data - png file
- Supplementary Data - png file
