# Supplementary figures and images for: OVA: integrating molecular and physical phenotype data from multiple biomedical domain ontologies with variant filtering for enhanced variant prioritization
Source: Bioinformatics. 2015 Aug 12;31(23):3822–9. doi: 10.1093/bioinformatics/btv473 (PMC4653395; doi:10.1093/bioinformatics/btv473)

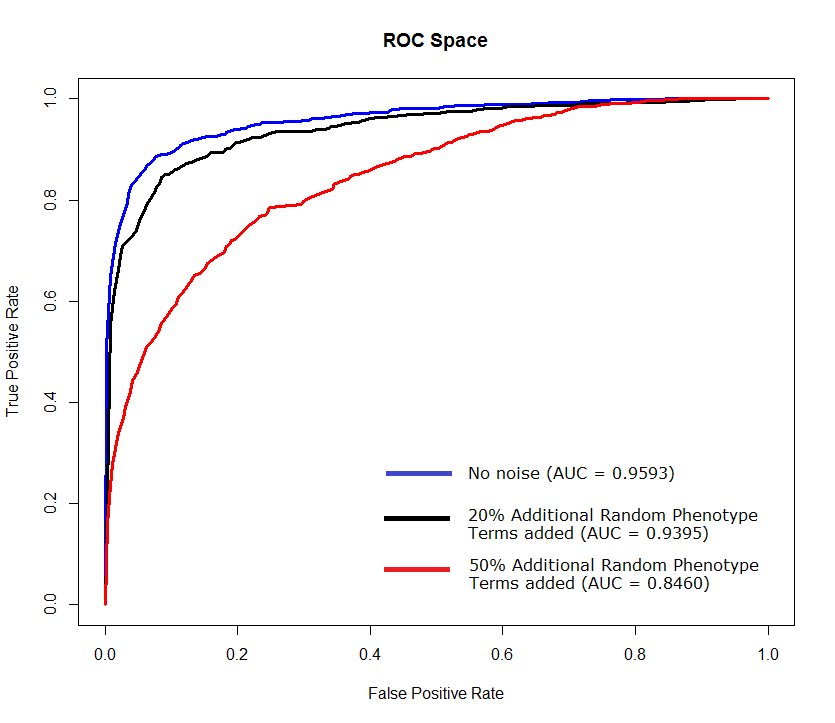

Supplement: Supplementary Data [file supp_btv473_SupplementaryFigure1A.png]

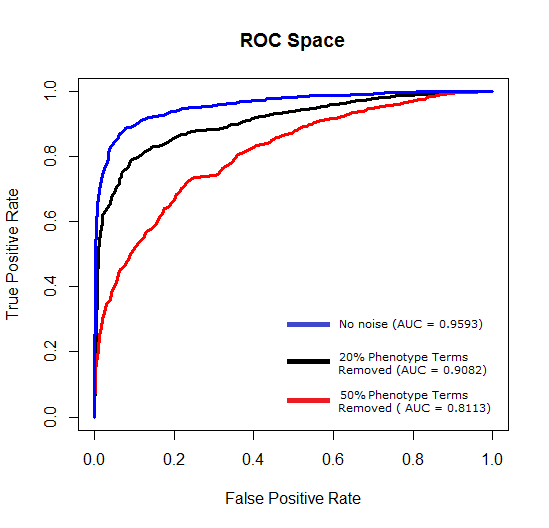

Supplement: Supplementary Data [file supp_btv473_SupplementaryFigure1B.png]

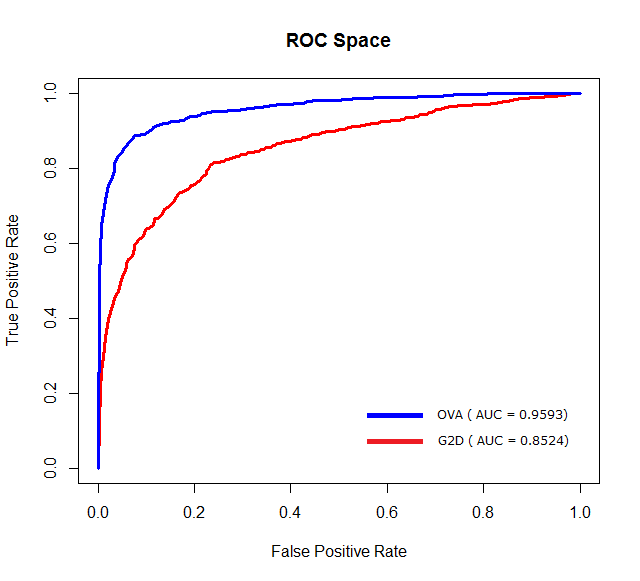

Supplement: Supplementary Data [file supp_btv473_SupplementaryFigure2.png]
